# Teachers Between a Rock and a Hard Place: Goal Conflicts Affect Teaching Motivation Mediated by Basic Need Satisfaction

**DOI:** 10.3389/fpsyg.2022.876521

**Published:** 2022-06-03

**Authors:** Julia Gorges, Phillip Neumann, Jan Christoph Störtländer

**Affiliations:** ^1^FB21 Institute of Educational Science, Philipps-University Marburg, Marburg, Germany; ^2^Faculty of Education Science, Bielefeld University, Bielefeld, Germany

**Keywords:** goal conflict, self-determination theory, basic needs, teaching motivation, structural equation modeling

## Abstract

Teaching is a highly demanding profession that requires handling multiple and potentially contradictory goals. Therefore, it is likely that teachers experience conflict between work-related goals on a daily basis. Intraindividual goal conflict may occur when individuals pursue multiple goals drawing on the same limited resources (resource-based goal conflict), or when two or more goals are incompatible in terms of goal attainment strategy or desired end states (inherent goal conflict). Because goal conflict is typically associated with negative effects such as attenuated motivation and wellbeing, teacher goal conflict may jeopardize teaching motivation. This cross-sectional study investigated the effects of in-service teachers’ (*N* = 302) goal conflicts on their autonomous (intrinsic and identified regulation) and controlled (introjected and extrinsic regulation) teaching motivation and tested the satisfaction of teachers’ basic need for autonomy, competence, and relatedness as mediators. In line with our hypotheses, results from structural equation modeling showed that frequently experiencing resource-based goal conflict leads to a lower satisfaction of the basic need for autonomy, which, however, was unrelated to teaching motivation. In contrast, frequently experiencing inherent goal conflict attenuates the satisfaction of the basic need for competence, which, in turn, positively predicted autonomous teaching motivation and negatively predicted extrinsic regulation. As expected, relatedness was not associated with the experience of goal conflict. The discussion focuses on differential effects of the two types of goal conflict on teaching motivation and on the relevance to expand research on teachers’ intraindividual goal conflicts.

## Introduction

Teaching is a highly demanding profession that requires significant psychological resources to sustain work motivation and engagement (Travers and Cooper, 1996; Kyriacou, 2001; Mansfield et al., 2012a; Dietrich, 2014; Embse et al., 2019). Studies show that a large proportion of teachers experience burnout at least once in their career; many of them leave the teaching profession before their official retirement (Byrne, 1991; Schwarzer et al., 2000; Dick and Wagner, 2001; Hakanen et al., 2006; Borman and Dowling, 2008). Prior to leaving, teachers may struggle with keeping up their motivation from day to day (Chang, 2009; Kelchtermans, 2017; Torenbeek and Peters, 2017; McInerney et al., 2018). Because teaching motivation is a key factor for high-quality teaching and teacher wellbeing (Watt et al., 2012; Nitsche et al., 2017; Ahn et al., 2021; Bardach and Klassen, 2021), teachers and students benefit from expanding our knowledge on teachers’ motivation. Understanding the factors that may jeopardize teachers’ motivation to teach—potentially to such an extent that teachers leave their job—is among the most urgent research topics (Dick and Wagner, 2001; Kyriacou, 2001; Embse et al., 2019).

From the perspective of self-determination theory (SDT; Deci and Ryan, 2004), it is important to distinguish between autonomous and controlled forms of motivation. Autonomous motivation—being motivated by internal factors such as joy or perceived importance of a task—is associated with positive outcomes such as higher wellbeing and achievement (Deci and Ryan, 2004; Gagné and Deci, 2005; Ahn et al., 2021; Bardach and Klassen, 2021). By contrast, controlled motivation—being motivated by reward and punishment—is associated with feeling pressured and stressed out (Pelletier et al., 2002). Several studies suggest that autonomous teaching motivation supports autonomous student motivation, student wellbeing, and academic achievement (Roth et al., 2007; Slemp et al., 2020; Ahn et al., 2021; Bardach and Klassen, 2021).

According to SDT, autonomous teaching motivation builds on the satisfaction of teachers’ basic psychological needs for autonomy, competence, and relatedness (Deci and Ryan, 2004; Slemp et al., 2020). We assume that, because teachers’ work conditions encompass many different tasks that need to be accomplished (e.g., preparing lessons, consulting with parents, and planning extracurricular activities), including contradictory tasks that are not reconcilable (e.g., incorporating novel learning content while covering the obligatory curriculum; Jones, 2003; Goddard et al., 2006; Mansfield et al., 2012a; Hutner et al., 2022), teachers pursue multiple and potentially conflicting goals on a daily basis. In turn, teachers’ experience of goal conflict may negatively affect the satisfaction of their basic needs and, in turn, their teaching motivation.

Against this background, the goal of the present study was to investigate how teachers’ experience of two types of goal conflict—resource-based and inherent goal conflict—may affect their teaching motivation. We propose that frequent experiences of goal conflict affect teachers’ satisfaction of the basic need for autonomy and competence, which, in turn, attenuate autonomous teaching motivation and increase controlled teaching motivation. To test our hypotheses, we surveyed in-service teachers about their experience of goal conflict, satisfaction of basic needs, and teaching motivation. Results of our study bear significance for understanding how intraindividual goal conflict, which is likely to arise from teachers working conditions, may jeopardize teacher motivation, thereby heightening the risk of attrition and burnout.

## Teaching Motivation From a Self-Determination Theory Perspective

In the light of multiple-goal pursuit on a daily basis, factors that sustain and develop teachers’ motivation and wellbeing are key to long-term teaching motivation and engagement in school. From the perspective of SDT, variations in the *quality* of motivation regulation constitute a key factor for performance and wellbeing (Deci and Ryan, 2000; Gagné and Deci, 2005; Ryan and Deci, 2020). Therefore, whether teachers engagement in teaching is driven by external pressure (extrinsic regulation of motivation), a sense of obligation (introjected regulation), because they perceive teaching as important (identified regulation), or because they enjoy teaching (intrinsic regulation), differentially affects teacher wellbeing, teaching performance, and long-term engagement in schools (Slemp et al., 2020).

Research documents that endorsement of autonomous (i.e., intrinsic and identified regulation) motivation, as opposed to controlled (i.e., introjected and extrinsic regulation) motivation, nourishes teacher wellbeing and job satisfaction, and promotes students’ wellbeing and achievement *via* motivating instructional practices (Guay et al., 2001; Pelletier et al., 2002; Roth et al., 2007; Niemiec and Ryan, 2009; Slemp et al., 2020). High autonomous teaching motivation leads teachers to putting more effort into supporting their students’ autonomy, leads to higher teacher wellbeing, and lower teacher stress, whereas high controlled teaching motivation leads teachers to be more controlling toward their students (Pelletier et al., 2002; Deci and Ryan, 2004; Milyavskaya and Koestner, 2011).

Contextual factors that affect teachers’ self-determined motivation are, for example, educational reforms; school leadership; resources for teaching; pressure from the school administration, colleagues, parents, and students; and their own personality, such as the need to meet certain performance standards (Byrne, 1991; Pelletier et al., 2002; Slemp et al., 2020; Hutner et al., 2022). According to SDT (Deci and Ryan, 2004), such contextual factors should differentially contribute to the satisfaction of basic psychological needs, which is considered the most proximal factor leading to autonomous rather than controlled motivation. The three basic needs postulated within the SDT framework encompass the need to experience oneself as agentic and autonomous (basic need for autonomy), the need to experience oneself as successful at challenging tasks and competent in attaining desired outcomes (basic need for competence), and the need to experience oneself as socially connected to other people (basic need for relatedness; Deci and Ryan, 2000; Roth, 2014). Previous research from other contexts has shown that the satisfaction of basic needs can act as a mediator between (the attainment of) goals and work values on the one hand and measures of (job) satisfaction and wellbeing on the other hand (Niemiec et al., 2009; Busque-Carrier et al., 2021).

Given the range of positive consequences of autonomous (teaching) motivation, many researchers have investigated its antecedents, particularly focusing on the role of basic need satisfaction (Roth, 2014; Slemp et al., 2020). Overall, substantial empirical evidence suggests that all basic needs contribute to teachers’ autonomous motivation and wellbeing in schools (Pelletier et al., 2002; Klassen et al., 2012) and higher education (Stupnisky et al., 2018). A meta-analysis revealed that satisfaction of the need for competence is slightly more important than that of the need for autonomy, and that satisfaction of the need for relatedness is least important (Slemp et al., 2020).

Consistent with the findings on teachers’ experience of competence, negative associations between teacher self-efficacy and teacher stress have been documented in cross-sectional (Bottiani et al., 2019) and longitudinal studies (Abele and Candova, 2007). Friedman (2000) and Brown (2012) point out that a lack of self-efficacy is a key predictor of burnout. Bridging external demands and individual emotions and cognitions, Pelletier et al. (2002) proposed that contextual factors may affect the satisfaction of basic needs, which, in turn, has the potential to promote autonomous teaching motivation and wellbeing.

## Goal Conflicts and Teaching Motivation

### Resource-Based and Inherent Goal Conflicts

From a psychological perspective, goals can be defined as “internal representations of desired states” (Austin and Vancouver, 1996, p. 338). Goals can be addressed on various levels of abstraction ranging from abstract, long-term goals (e.g., life goals; Pöhlmann and Brunstein, 1997) to very specific means goal systems (Kruglanski et al., 2002; Kelly et al., 2015). This study focused on mid-term to long-term goals that structure, energize, and shape people’s behavior and provide meaning in everyday life (Austin and Vancouver, 1996; Brunstein and Maier, 1996).

Goal conflict occurs when “a goal that a person wishes to accomplish interferes with the attainment of at least one other goal that the individual simultaneously wishes to accomplish” (Emmons et al., 1993, p. 531). Facing goal conflict may unleash new energy and heighten people’s motivation in a positive way (Gorges et al., 2014). However, it is typically associated with negative consequences for goal attainment and wellbeing (Emmons and King, 1988; Brunstein, 1993; Locke et al., 1994; Boudreaux and Ozer, 2013; Kelly et al., 2015; Gray et al., 2017). Therefore, we assume that teachers’ work-related goal conflict attenuates their autonomous motivation and wellbeing.

Teachers at public schools tackle a wide range of tasks on a daily basis (e.g., preparing lessons, supporting struggling students, talking to parents, administering exams, and contributing to school development; Byrne, 1991; Jones, 2003; Goddard et al., 2006; Mansfield et al., 2012a; Hutner et al., 2022). Excessive demands, strictly speaking, more goals than a teacher can handle at a time, is among the most frequently mentioned factors leading to teacher stress and burnout (Dick and Wagner, 2001; Fernet et al., 2013; Trépanier et al., 2013; Bottiani et al., 2019; Rajendran et al., 2020). Because teachers pursue multiple goals simultaneously, but their resources—for example, time and energy—are limited, we assume that teachers face *resource-based goal conflicts* on a regular basis (i.e., goal conflicts that occur because two or more goals use the same limited resources; Emmons and King, 1988; Riediger and Freund, 2004).

Resource-based goal conflicts can be addressed by various strategies. Tapping into additional resources (e.g., teaming up with colleagues), optimizing one’s action regulation, or reconsidering one’s priorities could help teachers to deal with resource-based goal conflicts. Handling multiple goals simultaneously may even lead to teachers channeling their energy to succeed in goal attainment, resulting in a positive affect (Gorges et al., 2014). Nevertheless, juggling multiple goals requires self-regulation competence and mental resources to manage time and resources in a manner that maximizes goal attainment and thereby minimizes failure to accomplish one’s goals (Muraven and Baumeister, 2000). These demands may lead to feeling overwhelmed by excessive work with little opportunity to proactively shape one’s work environment or deciding on daily tasks. Furthermore, teachers may feel limited in their freedom of action if they cannot solve the conflict the way they would like to. Therefore, in line with empirical findings documenting negative effects of resource-based goal conflicts on goal attainment and wellbeing (Boudreaux and Ozer, 2013; Kelly et al., 2015; Gray et al., 2017), these goal conflicts are likely to be a key factor for teacher stress and burnout (Dick and Wagner, 2001; Kyriacou, 2001; Embse et al., 2019).

Teachers may also experience *inherent goal conflicts.* Inherent goal conflicts occur because two or more goals involve incompatible goal attainment strategies (i.e., pursuing one goal means moving further away from another goal) or incompatible end states (e.g., one cannot be best friends with students and a stern teacher at the same time; (Helsper, 2004; Riediger and Freund, 2004; Segerstrom and Solberg Nes, 2006). For teachers, inherent goal conflicts may arise because they work in a contradictory environment, which is due—at least partly—to the structure of the pedagogical profession. To comply with their role as a teacher, teachers have to navigate “endemic uncertainties” (Lortie, 1975) or “antinomies” (Helsper, 2004), strictly speaking, contradictory requirements that simultaneously claim exclusive validity. For example, being a good teacher requires professional closeness to students but, simultaneously, it requires professional distance, to be able to grade students fairly. The concept of inherent goal conflict relates to self-discrepancies (i.e., discrepancies between the actual-self and the ought-self), which have been found to negatively affect wellbeing (Kelly et al., 2015).

Empirical research focusing on inherent goal conflicts is scarce (cf. Kelly et al., 2015; Gorges et al., 2017). Existing studies often address inherent goal conflicts that stem from avoidance goals clashing with approach goals (e.g., losing weight vs. enjoying dinner with friends). Nevertheless, in the light of the specific structure of teachers’ work environment and the various agents that set goals in pedagogical settings (e.g., head of school, government, parents, colleagues), we assume that teachers’ work-related goals may result in inherent goal conflicts on a regular basis. Remarkably, in a study by Segerstrom and Solberg Nes (2006), trained raters, who assessed interrelations of goals listed by study participants, reported more inherent goal conflicts than the participants themselves. Similarly, Riediger (2001) reported a substantial correlation between perceived resource-based and inherent conflicts for goal interrelations rated by study participants. Thus, people may not distinguish accurately between resource-based and inherent goal conflicts in everyday life.

Inherent goal conflicts—by definition—cannot be solved without abandoning one or more goals involved. Because school teachers often cannot choose not to pursue goals that conflict with other goals, teachers are at the mercy of the goal conflicts that are structurally created by their work context. Hence, teachers would feel less competent as they aspire to pursue all of their goals, but need to abandon some of the conflicting goals. Therefore, inherent goal conflict may contribute to teachers’ stress and burnout by fueling feelings of helplessness and incompetence (Friedman, 2000; Kyriacou, 2001; Chang, 2009).

### The Present Study: How Goal Conflicts May Affect Teaching Motivation

Goal conflict has predominantly negative effects on people’s experience and wellbeing (Boudreaux and Ozer, 2013; Gray et al., 2017), which presumably applies to teachers as well. Results from a study on teaching motivation of junior scientists at universities show that basic need satisfaction—satisfaction of the need for autonomy in particular—was associated with the occurrence of goal conflict and the experience of psychological strain due to goal conflict, whereas satisfaction of the basic need for competence was associated with autonomous teaching motivation (Esdar et al., 2016). Therefore, we propose that the negative relationship between conflicting goals and lower self-determined motivation is mediated by the satisfaction of basic psychological needs (Niemiec et al., 2009; Busque-Carrier et al., 2021), in particular, by diminishing perceptions of autonomy and competence.

Pursuing multiple goals generally limits an individual’s room for maneuver (Neal et al., 2017). The fact that teachers experience resource-based goal conflict highlights that they have too little time and energy to attain all their goals. Teachers may attempt to reduce the frequency of experiencing resource-based goal conflict by, for example, reorganizing their time or delegating tasks to other members of the staff (Böhm-Kasper et al., 2016; Paulsrud and Nilholm, 2020). However, because resources in schools are scarce and teachers cannot fall back on auxiliary staff, delegation probably does not work for teachers (Lortie, 1975; Tomasik and Freund, 2015; Goldan, 2021). Owing to resource constraints limiting teachers’ autonomy in managing goal conflict, teachers cannot solve the goal conflict the way they would like to, and continue to experience goal conflict despite their efforts to manage their goals. Feeling trapped between the overwhelming demands instigated by multiple goals that utilize the same resources, leaving little room for navigation, should attenuate teachers’ sense of autonomy. Therefore, we propose that the more frequently teachers experience resource-based goal conflict, the less autonomous they feel at work.

In contrast, inherent goal conflict cannot be resolved by applying strategies of self-regulation or reorganization. They persist, regardless of the strategies applied and the resources invested. People’s actions, despite their best efforts and increased resource investment, have limited impact on inherent goal conflict, which then hinder goal attainment (Riediger and Freund, 2004; Gorges et al., 2014). Because some goal conflicts are inherent in teaching, teachers may have to abandon conflicting goals and may doubt their professional competence when struggling while trying to satisfy all goals. Therefore, we assume that the more frequently teachers experience inherent goal conflict, the less competent they feel at work.

Overall, we suggest that the higher the frequency of goal conflicts encountered by teachers, the lower their perceived satisfaction of the basic needs for autonomy and competence. Low satisfaction of basic needs, in turn, will negatively affect teacher wellbeing (Emmons and King, 1988; Brunstein, 1993; Gagné and Deci, 2005) and result in controlled rather than autonomous teaching motivation (Deci and Ryan, 2000; Slemp et al., 2020). Because the basic need for relatedness does not seem to be closely associated to goal conflict, we did not hypothesize the satisfaction of the basic need for relatedness as a mediator of the effect of experiencing goal conflict. Nevertheless, to scrutinize these theoretical considerations, we assessed the role of relatedness as well.

Against this background, the goal of the present study was to investigate the effect of goal conflict on teachers’ motivation to teach. This is a significant departure from previous studies, which have almost exclusively focused on resource-based goal conflict (Gorges et al., 2017) and have rarely addressed teachers’ action goals (but see Mansfield et al., 2012b). We hypothesized that the frequency of experiencing goal conflict negatively affects teachers’ motivation to teach. Furthermore, we hypothesized that the satisfaction of teachers’ basic needs for autonomy and competence in the workplace mediates this link because goal conflict indicates that a teacher does not have the autonomy or competence to adequately deal with the many tasks they face every day. In particular, inherent goal conflict, for which teachers have no solution, should diminish their sense of competence, whereas juggling multiple goals that use the same resources should attenuate teachers’ perception of autonomy. Therefore, we tested the following hypotheses:

H1: The frequency of experiencing resource-based goal conflicts negatively predicts the teachers’ satisfaction of the basic need for autonomy, which, in turn, positively predicts autonomous forms of teaching motivation (intrinsic and identified regulation) and negatively predicts controlled forms of teaching motivation (extrinsic and introjected regulation).
H2: The frequency of experiencing inherent goal conflicts negatively predicts the teachers’ satisfaction of the basic need for competence, which, in turn, positively predicts autonomous forms of teaching motivation (intrinsic and identified regulation) and negatively predicts controlled forms of teaching motivation (extrinsic and introjected regulation).

## Methods

### Sample and Procedure

Cross-sectional data was derived from the third and fourth wave of a longitudinal study on the development of children with special educational needs in primary and secondary inclusive schools as well as special education schools in Germany. Regular and special education teachers were invited to participate in an online survey at the end of fourth grade and at the beginning of fifth grade, when their students were approximately 10–11 years old.

We constructed our sample by pooling teacher responses from these measurement points. Because most students transferred to secondary school after finishing fourth grade, only very few teachers were invited to participate twice. For those who did, data from the second measurement point was excluded from the analysis. The survey was conducted *via* Unipark (see Wild et al., 2017 for further details on the data collection process).^1^ From a total of 335 teachers who started the survey, all teachers who responded to at least one item of the measures considered here, were included in the analyses [*N* = 302; age: *M*(*SD*) = 45.5(10.5) years; 84.4% female; working at 90 different schools]. The majority worked at a primary school (*n* = 136), and others worked at special education schools (*n* = 99) and secondary schools (*n* = 67).

### Instruments

#### Resource-Based Goal Conflict and Inherent Goal Conflict

We assessed how frequently teachers experienced resource-based and inherent goal conflict using newly developed items presented in Supplementary Appendix 1 (Gorges et al., 2014). The measure used the item stem: “How often does it happen that you feel torn between ‘…’,” and participants were asked to fill the gap (‘…’) with the respective item ending referring to resource-based goal conflict (six items, e.g., “…lesson preparation and other activities?”), or referring to an inherent goal conflict (eight items, e.g., “…the goal of supporting a student and striving to further him/her?”). Responses were recorded on a 5-point Likert-type scale (1 = *Very rarely/never*, 2 = *rather rarely*, 3 = *sometimes*, 4 = *rather often*, 5 = *very often*). Internal consistency was good (see Table 1).

**TABLE 1:** Descriptive statistics, internal consistency, and bivariate correlations based on latent scale scores.

	*M* (*SD*)	α	(2)	(3)	(4)	(5)	(6)	(7)	(8)	(9)
(1) Inherent goal conflict	2.69 (0.73)	0.83	0.62*	–0.40*	−0.40*	–0.07	0.30*	0.24*	–0.09	−0.18*
(2) Resource-based goal conflict	2.94 (0.71)	0.78		–0.32*	−0.46*	–0.08	0.18*	0.08	–0.05	–0.09
(3) Competence	3.07 (0.44)	0.62			0.69*	0.58*	−0.37*	–0.12	0.49*	0.57*
(4) Autonomy	2.72 (0.61)	0.76				0.34*	−0.17*	–0.07	0.13*	0.17*
(5) Relatedness	3.49 (0.42)	0.69					−0.24*	0.01	0.44*	0.40*
(6) Extrinsic regulation	1.78 (0.60)	0.83						0.59*	–0.06	–0.13
(7) Introjected regulation	2.63 (0.62)	0.67							0.40*	0.16*
(8) Identified regulation	3.66 (0.40)	0.78								0.86*
(9) Intrinsic regulation	3.61 (0.43)	0.79								−

Responses regarding the frequency of experiencing of goal conflict were recorded on a scale from 1 to 5, other responses were recorded on a scale from 1 to 4; *p < 0.05.

#### Extrinsic, Introjected, Identified, and Intrinsic Regulation of Teaching Motivation

Regulation of teaching motivation was measured using a four-dimensional measure adapted from Stegmüller (2012), which was based on Müller et al. (2009). The item stem was laid out as follows: “When I’m committed as a teacher, that’s because….” Each subscale included four items to complement the item stem, which concluded in the measure comprising 16 items in total. For example, extrinsic regulation of teaching motivation was assessed using the item “When I’m committed as a teacher, that’s because otherwise I will have problems with the principal,” introjected regulation was assessed using the item “When I’m committed as a teacher, that’s because I would have a bad conscience if I did not try hard enough,” identified regulation was assessed using the item “When I’m committed as a teacher, that’s because it is important to me that the students learn something,” and intrinsic regulation was assessed using the item “When I get involved as a teacher, that’s because I have fun teaching students.” Responses were recorded on a 4-point Likert-type scale (1 = *does not apply at all*, 2 = *rather does not apply*, 3 = *rather applies*, 4 = *fully applies*). Internal consistency was good (see Table 1).

#### Satisfaction of the Basic Needs for Autonomy, Competence, and Relatedness

Satisfaction of the basic needs for experiencing autonomy (four Items, e.g., “I have the feeling that I can strongly influence how I organize my work.”), competence (four items, e.g., “On most days, I go home feeling like I accomplished a lot”), and relatedness (four items, e.g. “I am important to the people in my work environment.”) was measured using items adapted from Hanfstingl et al. (2010). Responses were recorded on a 4-point Likert-type scale (1 = *does not apply at all*, 2 = *rather does not apply*, 3 = *rather applies*, 4 = *fully applies*). Internal consistency was at least acceptable (see Table 1).

### Statistical Analyses

We conducted our analyses based on latent variable modeling using R (R Development Core Team, 2008) and the lavaan package (Rosseel, 2012). Using latent variable modeling allowed us to measure the constructs of interest without measurement error (i.e., due to the fact that measurement error can be captured by the residuals specified in the model). Because our participants came from many different schools with only a few teachers coming from the same school, we did not consider school as a second level in our analyses. The percentage of missing value per variable ranged from 0 to 1.32%. We evaluated model fit based on the χ^2^/*df*-ratio, the comparative fit index (CFI; acceptable fit indicated by CFI > 0.90, good fit indicated by CFI > 0.95), the root mean square error of approximation (RMSEA; acceptable fit indicated by RMSEA < 0.08, good fit indicated by RMSEA < 0.05), and the standardized root mean squared residual (SRMR; acceptable fit indicated by SRMR < 0.08, good fit indicated by SRMR < 0.05; Schermelleh-Engel et al., 2003). Measurement models were accepted as at least satisfactory with significant factor loadings of λ > 0.30 (*p* < 0.05).

We started our analyses by conducting confirmatory factor analyses (CFA) to test our measurement models for goal conflict, satisfaction of basic needs, and teaching motivation. To account for missing data, we used the full information maximum likelihood estimation with robust (Huber-White) standard errors and a test statistic that is (asymptotically) equal to the Yuan–Bentler test statistic. To assess the appropriateness of the measurement models, we inspected the model fit and compared a one-factor model with a model distinguishing the theoretically assumed factors of each construct by comparing the AIC (Akaike information criterion) and the BIC (Bayesian information criterion; smaller values indicate better fit), and conducting χ^2^-difference tests.

To test our hypotheses, we used structural equation modeling (SEM) with Maximum Likelihood estimation, which is by default based on the biased sample covariance matrix, and bootstrapping of *p*-values (test = “bootstrap”) with 10,000 iterations. First, to avoid multicollinearity issues, we specified three separate models, one for each mediator (i.e., autonomy, competence, and relatedness). We evaluated the statistical significance and effect sizes of path coefficients predicted by our hypotheses. More specifically, we tested frequency of experiencing resource-based goal conflicts as a predictor of the satisfaction of the basic need for autonomy, which, in turn was hypothesized to predict teaching motivation. Similarly, we tested frequency of experiencing inherent goal conflicts as a predictor of the satisfaction of the basic need for competence, which, in turn was hypothesized to predict teaching motivation. Finally, to cover all basic needs postulated by SDT, we tested frequency of experiencing goal conflicts as a predictor of the satisfaction of the basic need for relatedness, which we did not hypothesize, and possible effects of relatedness on regulation of teaching motivation. In addition, we estimated the size and significance of the hypothesized indirect effects, that is, frequency of experiencing inherent goal conflict predicting regulation of teaching motivation mediated by satisfaction of the basic need for competence, and frequency of experiencing resource-based goal conflict predicting regulation of teaching motivation mediated by satisfaction of the basic need for autonomy. Resource-based and inherent goal conflicts on the one hand and the four dimensions of teaching motivation on the other hand were allowed to correlate.

Next, to assess the interplay of all variables, we specified a full model considering all three basic needs as mediators simultaneously. In this model, the latent variables indicating satisfaction for the basic need for autonomy, competence, and relatedness, were allowed to correlate as well (Model 1). Lastly, because we did not predict relatedness to be a relevant mediator, we specified a mediation model including all three basic needs as mediators but fixed the paths between frequency of experiencing inherent and resource-based goal conflict and relatedness, and relatedness and regulation of teaching motivation, to zero (Model 2). Again, we estimated the sizes and significance of the hypothesized indirect effects.

We predicted that the frequency of experiencing resource-based goal conflicts affects autonomous and controlled teaching motivation mediated by the satisfaction of the basic need for autonomy, and that the frequency of experiencing inherent goal conflicts affects autonomous and controlled teaching motivation mediated by the satisfaction of the basic need for competence.

## Results

### Preliminary Analyses: Measurement Models, Descriptive Statistics, and Correlations

**FIGURE 1:**
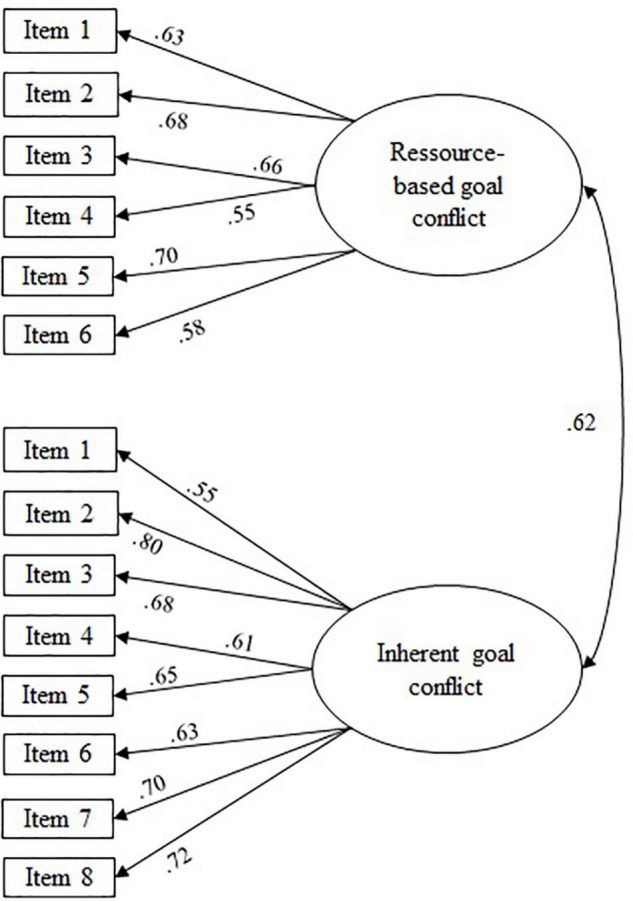
Path coefficients from confirmatory factor analyses: two-factor model of frequency of experiencing goal conflict. Figure depicts standardized loadings; all loadings and correlations are significant (*p* < 0.05); correlations between latent variables were specified as residual error correlations.

**FIGURE 2:**
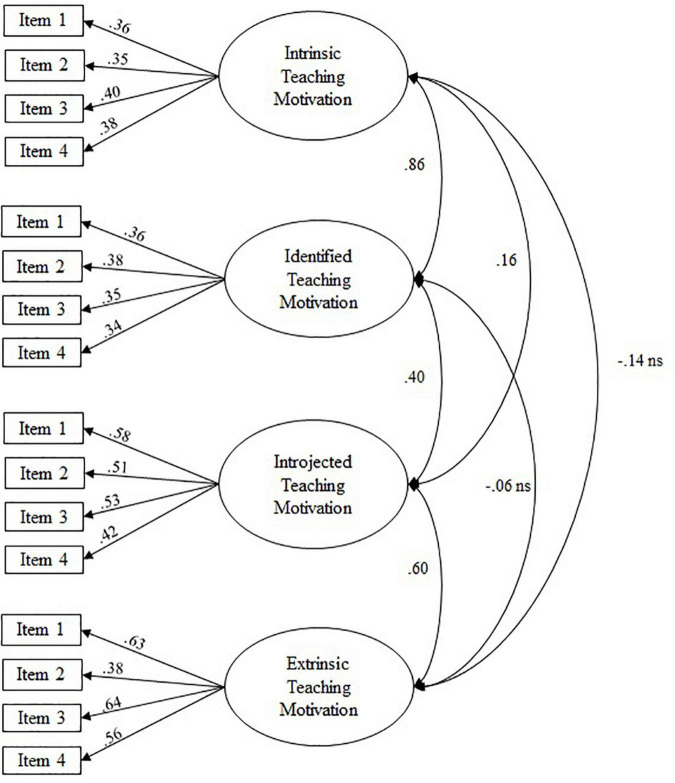
Path coefficients from confirmatory factor analyses: four-factor model of teaching motivation. Figure depicts standardized loadings; all loadings and correlations are significant (*p* < 0.05) if not otherwise stated; correlations between latent variables were specified as residual error correlations.

As expected, distinguishing multiple factors according to theoretical considerations was necessary to obtain an acceptable fit of the measurement models (see Table 2, Measurement Models). That is to say, a two-factor model distinguishing between resource-based and inherent goal conflict fit significantly better [χ^2^(1) = 113.16, *p* < 0.05] than a one-factor model without this distinction (see Figure 1). Similarly, the four-factor model distinguishing four qualities of teaching motivation (see Figure 2) fit significantly better than a one-factor model without any distinctions [χ^2^(1) = 712.88, *p* < 0.05], or a two-factor model distinguishing only between autonomous and controlled forms of teaching motivation [χ^2^(5) = 114.76, *p* < 0.05].

**TABLE 2:** Model fit for measurement models.

Model	χ ^2^	*df*	*p*	CFI	RMSEA [90% CI]	SRMR	AIC	BIC
**Goal conflict**								
1-factor model	244.060	77	<0.001	0.82	0.085 [0.075–0.096]	0.079	11432.27	11535.88
2-factor model	130.897	76	<0.001	0.94	0.049 [0.036–0.062]	0.055	11282.39	11389.70
**Basic need satisfaction**								
1-factor model	335.943	54	<0.001	0.63	0.132 [0.119–0.145]	0.097	6680.26	6813.48
3-factor model	148.073	51	<0.001	0.87	0.080 [0.066–0.094]	0.059	6484.72	6629.03
Adj. 3-factor model^a^	121.101	52	<0.001	0.91	0.067 [0.052–0.081]	0.063	6453.70	6594.32
**Teaching motivation**								
1-factor model	976.157	104	<0.001	0.39	0.177 [0.167–0.187]	0.221	8852.17	8970.37
2-factor model	260.273	103	<0.001	0.90	0.077 [0.065–0.089]	0.101	8075.30	8197.20
4-factor model	145.513	98	0.001	0.99	0.043 [0.027–0.057]	0.067	7591.15	8091.51
**Mediation models**								
Model autonomy	786.591	507	<0.001	0.91	0.043 [0.037–0.049]	0.062	21384.36	21708.52
Model competence	779.401	506	<0.001	0.91	0.043 [0.037–0.049]	0.060	21001.73	21329.27
Model relatedness	743.700	506	<0.001	0.92	0.040 [0.034–0.046]	0.058	20742.16	21069.70
Model 1	1125.052	784	<0.001	0.91	0.038 [0.033–0.043]	0.059	25743.97	26341.35
Model 2	1182.379	790	<0.001	0.90	0.041 [0.036–0.046]	0.061	25131.41	25547.27

^a^ model with adjusted autonomy subscale: negative multiplier added to negatively phrased items to facilitate interpretation (i.e., higher values reflect higher satisfaction of the basic need for autonomy) and error correlation allowed between negatively phrased items; Model Autonomy/Competence/Relatedness: Frequency of experiencing inherent and resource-based goal conflict predict extrinsic, introjected, identified and intrinsic regulation partially mediated by the satisfaction of the basic need for autonomy/competence/relatedness; Model 1: Mediation model with all three basic needs; Model 2: Model 1 with autonomy and competence as mediators; *p < 0.05.

**FIGURE 3:**
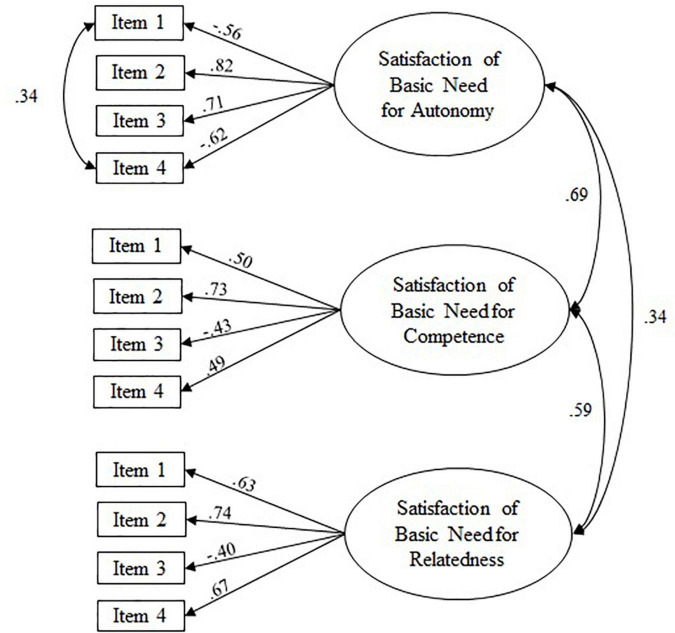
Path coefficients from confirmatory factor analyses: three-factor model of satisfaction of the basic needs for autonomy and competence. Figure depicts standardized loadings; all loadings and correlations are significant (*p* < 0.05); correlations between latent variables and indicators were specified as residual error correlations.

Regarding basic need satisfaction, the three-factor model fitted significantly better [χ^2^(3) = 187.87, *p* < 0.05] than the one-factor model. Because fitting the model produced positive loadings of the two negatively phrased items and negative loadings of the positively phrased items, our initial model resulted in a negatively coded latent variable, meaning, high values on the latent variable autonomy reflected low satisfaction of the basic need for autonomy. To facilitate interpretation of results, we decided to add negative multipliers of the magnitude of the loadings from the initial three-factor model to the estimated loadings of negatively phrased items to tip the item loadings into the opposite direction, resulting in positive loadings of the positively phrased items. Thus, the latent variable reflected the satisfaction of the basic need for autonomy. In addition, the CFI of the three-factor model was below the cut-off value of 0.90 for acceptable fit, indicating that correlations in the data were not reflected by the model specification. Therefore, we specified an error correlation between the two negatively phrased items of the autonomy scale (*r* = 0.34, *p* < 0.05) to achieve a good model fit (see Figure 3). The final measurement models showed at least an acceptable model fit with substantial and significant factor loadings (λ > 0.40; *p* < 0.05). Therefore, all latent variables could be used as planned in the subsequent analyses.

Table 1 summarizes the descriptive statistics and correlations derived from latent CFAs. Overall, the frequency of experiencing goal conflict ranged slightly below the absolute scale mean of three indicating that teachers “sometimes” experience goal conflict. Ratings regarding autonomy, competence, and relatedness tended toward a high satisfaction of basic needs. Teaching motivation was clearly autonomous rather than controlled.

Bivariate correlations were obtained from the latent CFAs. The correlations between the frequencies of experiencing resource-based and inherent goal conflict, and between the satisfaction of the basic need for autonomy and competence, were positive and strong, whereas relatedness correlated somewhat weaker with autonomy and competence. Resource-based goal conflict and inherent goal conflict were negatively associated with autonomy and competence and unrelated to relatedness. Competence correlated strongly and negatively with extrinsic regulation and strongly and positively with identified and intrinsic regulation; autonomy correlated substantially weaker with teaching motivation, but the correlations were in the expected direction. Relatedness correlated weakly but significant with extrinsic, identified and intrinsic regulation. Interestingly, inherent goal conflict appears to be more strongly associated to teaching motivation than resource-based goal conflict.

### Mediation Models

**FIGURE 4:**
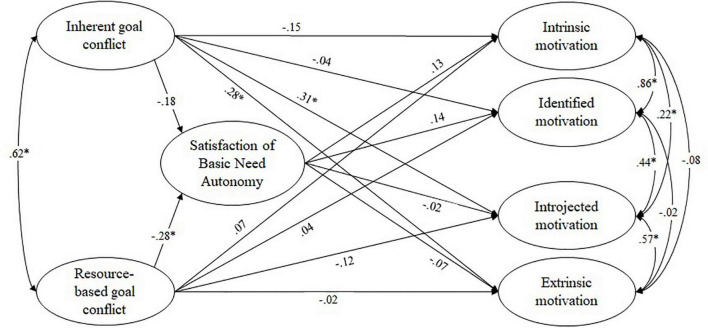
Path coefficients from structural equation modeling: frequency of experiencing goal conflict affecting teaching motivation *via* the satisfaction of the basic need for autonomy. Figure depicts standardized beta weights, correlations between latent variables were specified as residual error correlations; *p < 0.05.

The structural equation models showed at least an acceptable model fit (see Table 2). Path coefficients and correlations from the separate models for autonomy, competence, and relatedness, are depicted in Figures 4,– 6. Correlations were in line with the bivariate correlations reported above. We first looked at the satisfaction of the basic need for autonomy as a mediator (see Figure 4). As expected, a higher frequency of experiencing resource-based goal conflict predicted a lower satisfaction of the need for autonomy; the path between inherent goal conflicts and autonomy was not statistically significant. However, against expectations, the satisfaction of the need for autonomy did not significantly affect regulation of teaching motivation, and indirect effects were not significant. In addition, the results revealed a positive and direct effect of inherent goal conflict on introjected and extrinsic regulation of teaching motivation.

**FIGURE 5:**
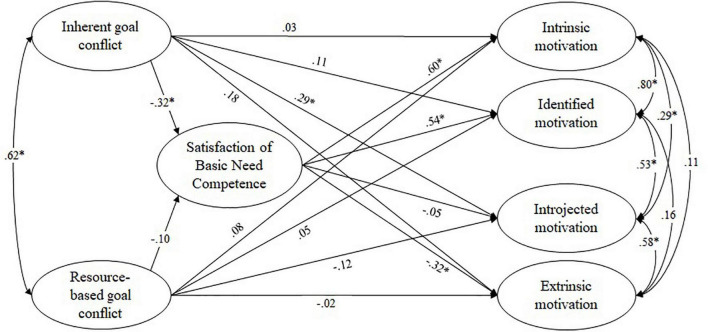
Path coefficients from structural equation modeling: frequency of experiencing goal conflict affecting teaching motivation via the satisfaction of the basic need for competence. Figure depicts standardized beta weights, correlations between latent variables were specified as residual error correlations; *p < 0.05.

Turning to competence as a mediator (see Figure 5), results showed that, in line with our hypotheses, a higher frequency of experiencing inherent goal conflict predicted a lower satisfaction of the need for competence, whereas the path between resource-based goal conflict and competence was not statistically significant. Regarding teaching motivation, satisfaction of the basic need for competence strongly and positively predicted identified and intrinsic regulation, and negatively predicted extrinsic regulation. The indirect effect of the frequency of experiencing inherent goal conflict on regulation mediated by satisfaction of the basic need for competence was significant for intrinsic (β = –0.19, *p* < 0.05), identified (β = –0.17, *p* < 0.05), and extrinsic regulation (β = 0.10, *p* < 0.05). The direct path between inherent goal conflict and extrinsic regulation found in the previous model was no longer significant, hence satisfaction of the basic need for competence completely mediated this effect. However, the direct effect of inherent goal conflict on introjected regulation could be documented in this model as well.

**FIGURE 6:**
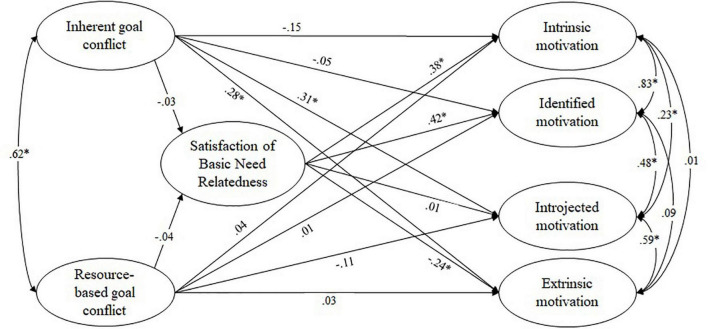
Path coefficients from structural equation modeling: frequency of experiencing goal conflict affecting teaching motivation via the satisfaction of the basic need for relatedness. Figure depicts standardized beta weights, correlations between latent variables were specified as residual error correlations; *p < 0.05.

In line with our expectations, the satisfaction of the basic need for relatedness was not predicted by frequency of experiencing goal conflict. It did, however, significantly predict intrinsic, identified, and extrinsic regulation when considered separately (see Figure 6).

Our final step was to assess the interplay of all variables in two comprehensive models (Model 1 considering all three basic needs as mediators; Model 2 with paths between frequency of experiencing goal conflict and relatedness, and relatedness and regulation, fixed to zero). Model fit indicated that considering relatedness did not contribute to the overall model fit: The model fit hardly changed when we fixed paths to zero, and none of these paths were significant (see Table 3).

**TABLE 3:** Path coefficients for the mediation model including the satisfaction of the basic need for autonomy, competence and relatedness (Model 1) and autonomy and competence (Model 2), respectively, as mediators.

Paths	Model 1	Model 2
**Direct effects**		
**Autonomy <=**		
Inherent goal conflict	–0.18	–0.18
Resource-based goal conflict	–0.28*	–0.28*
**Competence <=**		
Inherent goal conflict	–0.30*	–0.30*
Resource-based goal conflict	–0.09	–0.07
**Relatedness <=**		
Inherent goal conflict	–0.03	0.00
Resource-based goal conflict	–0.03	0.00
**Extrinsic regulation <=**		
Autonomy	0.23	0.28*
Competence	–0.39*	–0.51*
Relatedness	–0.10	0.00
Inherent goal conflict	0.21*	0.18
Resource-based goal conflict	0.03	0.04
**Introjected regulation <=**		
Autonomy	0.03	0.01
Competence	–0.10	–0.03
Relatedness	0.06	0.00
Inherent goal conflict	0.29*	0.30*
Resource-based goal conflict	–0.11	–0.12
**Identified regulation <=**		
Autonomy	–0.35*	–0.46*
Competence	0.61*	0.87*
Relatedness	0.20	0.00
Inherent goal conflict	0.07	0.13
Resource-based goal conflict	–0.04	–0.06
**Intrinsic regulation <=**		
Autonomy	–0.42*	–0.49*
Competence	0.78*	0.91*
Relatedness	0.09	0.00
Inherent goal conflict	–0.01	0.03
Resource-based goal conflict	–0.02	–0.04
**Indirect effects**		
**Extrinsic regulation <=**		
Inherent goal conflict *via* competence	0.12	0.16*
Resource-based goal conflict *via* autonomy	–0.06	–0.08
**Introjected regulation <=**		
Inherent goal conflict *via* competence	0.03	0.01
Resource-based goal conflict *via* autonomy	–0.01	–0.01
**Identified regulation <=**		
Inherent goal conflict *via* competence	–0.19*	–0.26*
Resource-based goal conflict *via* autonomy	0.10	0.13*
**Intrinsic regulation <=**		
Inherent goal conflict *via* competence	–0.24*	–0.27*
Resource-based goal conflict *via* autonomy	0.12	0.13*

Table depicts standardized path coefficients with bootstrapped p-values; Model 1: frequency of experiencing inherent and resource-based goal conflict predict extrinsic, introjected, identified and intrinsic regulation of teaching motivation partially mediated by the satisfaction of the basic need for autonomy, competence, and relatedness; Model 2: Model 1 with autonomy and competence as mediators, for further description see text; * p < 0.05.

Regarding the hypothesized mediation effects, the frequency of experiencing inherent goal conflicts, but not resource-based goal conflicts, significantly predicted the satisfaction of the basic need for competence in both models, which, in turn, significantly predicted extrinsic, identified, and intrinsic regulation. In Model 1 and Model 2, indirect effects of frequency of experiencing inherent goal conflicts on identified and intrinsic regulation were significant; in Model 2, the indirect effect on extrinsic regulation was significant as well.

Compared to the competence-model reported earlier, the effects of the satisfaction of the basic need for competence on the regulation were markedly stronger. In addition, the effect of the satisfaction of the basic need for autonomy was stronger and significant in this model, but had reversed signs. Overall, these results point to a negative suppression effect of autonomy (MacKinnon et al., 2000): Autonomy correlated strongly with competence, but correlated only weakly with regulation. Therefore, considering autonomy in the model appears to capture residual variance from the independent variable “competence,” thereby increasing the effect of the satisfaction of the basic need for competence on regulation, whereas the effect of the satisfaction of the basic need for autonomy on regulation cannot be interpreted. The results of Model 2 also include significant indirect effects of frequency of experiencing resource-based goal conflicts on identified and intrinsic regulation mediated by the satisfaction of the basic need for autonomy.

Lastly and consistent with the results from the separate mediation models, both Model 1 and Model 2 revealed a significant direct effect of frequency of experiencing inherent goal conflicts on introjected regulation, which was not predicted by any of the basic needs.

## Discussion

The present study aimed to investigate the effect of teachers’ experience of inherent and resource-based goal conflict on the autonomous and controlled forms of teaching motivation. The satisfaction of the basic needs for autonomy and competence, respectively, were investigated as mediating variables. Results show that frequently experiencing inherent goal conflict attenuated teachers’ perceptions of being competent, which, in turn, positively predicted intrinsic and identified regulation of teaching motivation and negatively predicted extrinsic regulation of teaching motivation. That is to say, frequently experiencing inherent goal conflict may lead to less autonomous and more controlled teaching motivation, which is associated with negative effects on teacher wellbeing and student motivation (Slemp et al., 2020; Ahn et al., 2021; Bardach and Klassen, 2021). In addition, frequently experiencing inherent goal conflict contributed directly to introjected regulation of teaching motivation. Results also showed that the more frequent teachers experience resource-based goal conflict, the lower their satisfaction of the basic need for autonomy, which, however, was unrelated to the regulation of teaching motivation. In line with expectations, frequency of experiencing goal conflict was unrelated to the satisfaction of the basic need for relatedness.

### Teachers’ Goal Conflicts and Teaching Motivation

Looking at the descriptive statistics for our sample, the average frequency of experiencing inherent goal conflict was lower than expected. Most mean values ranged around the response option *sometimes*. The perception of an inherent goal conflict, however, may be obscured when teachers try to pursue both conflicting goals regardless of the inherent nature of the conflict, which may lead to the perception of resource constraints rather than awareness of an inherent goal conflict. The average frequency of experiencing resource-based goal conflict, in contrast, was higher with most mean values ranging around the response option *rather often*, which is consistent with previous findings that indicate teachers’ high job demands based on an overwhelming workload (Travers and Cooper, 1996; Goddard et al., 2006; Mansfield et al., 2012a).

As expected, the more frequent teachers experience resource-based goal conflict, the lower the satisfaction of their need to experience themselves as autonomous, whereas the more frequent teachers experience inherent goal conflict, the lower the satisfaction of their need to experience themselves as competent. In other words, pursuing inherently conflicting goals seems to diminish teachers’ sense of competence. However, it remains unclear whether this effect would occur without teachers being aware of the inherent nature of the goal conflict because we explicitly asked to rate a situation depicting inherent goal conflict. The strong association between inherent goal conflict and autonomous teaching motivation mediated by the satisfaction of the need to feel competent suggests that frequently experiencing inherent goal conflict jeopardized desirable forms of teaching motivation more than frequently experiencing resource-based goal conflict. In the light of these findings and empirical evidence pointing to negative effects of context conditions (e.g., educational reforms) on teacher stress (Byrne, 1991; Hutner et al., 2022), which may results in contradicting demands, inherent goal conflict appears to be a relevant construct to explain adverse effects of external conditions on individual motivation and wellbeing.

The strong correlation between both types of goal conflict suggests that they co-occur and that the distinction between the two types is not always clear-cut for those who experience goal conflict (Segerstrom and Solberg Nes, 2006). Assuming that the awareness of inherent goal conflict, in particular, needs a certain degree of reflective action, goal conflicts may present themselves as resource based at first sight and may be recognized as inherent only later. In addition, teachers may even tend to reframe an inherent goal conflict as a resource-based goal conflict when they heavily invest resources into pursuing both goals, either because the futility of this endeavor is obscured or because they feel that they still need to try to work toward both goals (Author, 2020). Rationally, it seems most sensible to adjust one’s goal pursuit strategies to the type of goal conflict because different strategies may be most promising, and acknowledging an inherent goal conflict may relieve the pressure to succeed, leading to a more pragmatic approach to managing goal conflict. For this approach, however, recognition of the type of goal conflict is imperative.

A lack of satisfaction of the basic needs for competence and autonomy typically leads to more controlled motivation and less autonomous motivation and thereby precludes any positive consequences of autonomous motivation for teachers and students (Slemp et al., 2020). Against expectations, the basic need for autonomy did not predict autonomous motivation in our study. Instead, we found a strong association between feeling competent and endorsing autonomous motivation, which emphasizes the need to investigate the satisfaction of the basic need for competence. In addition, future research should focus on the link between the basic need for competence and self-efficacy to better understand how these constructs overlap and how, if at all, measures to support teacher self-efficacy have similar effects as satisfying the basic need for competence.

Our results also showed a direct effect of the frequency of experiencing inherent goal conflict on introjected regulation of teaching motivation. This finding suggests a lack of progress toward goals that are set by external agents but still stimulate teachers’ internal systems of reward (e.g., living up to one’s obligations) and sanctions (e.g., feeling guilty). Estimating the level of self-concordance for goal pursuit may enable a deeper understanding of the cognitive processes at work (Gorges et al., 2014).

Previous research that relates to goal conflict focused on the role of autonomous motivation as a predictor of experiencing conflicts between two or more life domains such as work and family, education and social relations, or education and leisure (Senécal et al., 2001, 2003; Ratelle et al., 2005). Results from three cross-sectional studies reveal that higher levels of autonomous motivation predict lower levels of conflict and, in turn, adverse outcomes (e.g., procrastination, emotional exhaustion). Thus, contrasting our approach, this research emphasizes a lack of autonomous motivation as prerequisite of experiencing conflict, whereas we argue that experiencing goal conflict leads to attenuated autonomous motivation by way of reducing the satisfaction of basic needs. We base our line of arguments on the processes taking place when school develop and react to context conditions such as educational reforms and policies that need to be implemented by teachers. Because our study, just as the three studies from Senecal, Ratelle, and colleagues, were cross-sectional, we cannot determine the direction of effects at this time. Considering our results and the results from previous research, reciprocal effects seem likely. That is to say, we assume that goal conflicts inflicted by contextual factors lead to attenuated levels autonomous motivation, and individuals’ level of autonomous motivation affects how they experience their environment including goal conflicts. Future research needs to unveil the interplay of goal conflict and motivation in various contexts.

### Limitations

Several limitations of our study should be acknowledged when interpreting the findings. First and as mentioned above, because the data were derived from a cross-sectional study, our results do not warrant causal conclusions. We argue that frequently experiencing goal conflict affects the satisfaction of the basic needs for autonomy and competence, which is a plausible mechanism regarding existing literature on the effect of educational reforms (Byrne, 1991; Hutner et al., 2022). Educational reforms change teachers’ work conditions and, in turn, should affect teachers’ work-related goals and goal conflicts. Based on our data, however, we cannot rule out that the satisfaction of the basic needs for autonomy and competence (also) affect teachers’ subjective perceptions of goal conflicts. That is to say, maybe teachers with low need satisfaction perceive their work to entail more goal conflicts than teachers whose basic needs are satisfied. Therefore, future research should set out to investigate the relation between experiencing goal conflict and satisfaction of basic needs using longitudinal data or experimental approaches.

We assessed the experience of goal conflict in a standardized way; therefore, the goals we used may not be identical to the goals our respondents actually set for themselves. We used newly developed scales to assess the frequency of experiencing goal conflict, which showed good factor validity and internal consistency. The scales’ discriminant and convergent validity, however, remains to be assessed more comprehensively in future research.

In addition, using rather abstract goals may make it difficult for participants to link the goal conflict specified in the item to the specific situations in which they experience a goal conflict, which would typically be a conflict between means (Kruglanski et al., 2002; Gorges et al., 2017). Therefore, future studies could implement an idiographic–nomothetic approach (Gorges et al., 2014), which would allow data collection on participants’ real-life goals. Ideally, the experience and consequences of goal conflict should be studied using a longitudinal design that is close to participants’ day-to-day life, such as experience sampling or diary studies (Riediger and Freund, 2004; Klusmann et al., 2021).

Our analytic approach relied on a sufficient sample size to yield valid results. To evaluate the statistical power of our study, we assessed under which conditions our sample size would have been recommended in *a priori* power analyses (α = 0.05, β = 0.80) on the basis of the number of indicators and latent variables in the single mediator models. According to Soper (2021), our sample size was sufficient to detect moderate effects (β = 0.24). Hence, we argue that our study has been fit to detect practically relevant effects.

Our study focused on the satisfaction of the basic needs for autonomy and competence because, theoretically, we argued that these two basic needs would be more closely associated with goal conflict than the need for relatedness. Because research has shown that teachers also need to feel socially related to people at work (Klassen et al., 2012), we considered relatedness as well, but did not find an association between relatedness and the frequency of experiencing goal conflict.

Regarding the assessment of basic need satisfaction, the error correlation that was allowed in the specification of the measurement model of the autonomy subscale may limit the generalizability of the results presented here. Therefore, we urge researchers to replicate and scrutinize our findings.

Extending the scope of goal-related research on teaching and teacher education, future research should consider both goal-related and personal-related characteristics that may contribute to differential effects of goal conflict on teachers’ behavior and wellbeing. For example, dispositional optimism, mindfulness, and tolerance of uncertainty have been found to affect how people perceive their goal systems and react to goal conflict or motivational interference (Segerstrom and Solberg Nes, 2006). Goal self-concordance and goal commitment have been emphasized as important goal characteristics in conflicting situations (Locke et al., 1994; Gorges et al., 2014). Furthermore, additional sources that fuel basic need satisfaction should be considered for a more complete picture of how goal conflict — in conjunction with other factors—affects the regulation of teaching motivation.

### Outlook

In sum, investigating teachers’ actions, perceptions, and emotions from a goal theory perspective seems to be a promising approach to reconcile scattered research on teachers’ motivation, emotions, wellbeing and performance. The pattern of our results suggests that the distinction between resource-based and inherent goal conflict will prove important in future studies. Because ours is the first study—to the best of our knowledge—to address this distinction explicitly (Gorges et al., 2017), further research is needed to scrutinize and extend our findings. Nevertheless, we did find that (a) the experience of inherent vs. resource-based goal conflict can be separated empirically, (b) the experience of both types of goal conflict is strongly correlated, and (c) each type has unique effects on teachers’ satisfaction of basic needs, and, consequently, the regulation of teaching motivation. Considering the documented relation to the satisfaction of basic needs, effects on teachers’ wellbeing also seem likely. Future research should also take into account possible effects of teachers’ goal attainment (Niemiec et al., 2009) and factors that may affect teachers choice of action goals, which may have implications for their motivation and wellbeing (Milyavskaya et al., 2014).

Goals directing teachers’ behavior is a complex albeit understudied field of research to date. As our study is among the rare studies that examine teachers’ work-related goals and goal conflict from an action regulatory perspective (Dick and Wagner, 2001), the present findings make a critical contribution to the literature. Future research exploring the prevalence of teachers’ goal conflict and how goal conflict affects key outcomes beyond teaching motivation (e.g., wellbeing, burnout, and intention to leave as well as teaching performance) is expected to further our understanding of teachers’ goal conflict. Nevertheless, research from this perspective is still in its early stages.

## Data Availability Statement

The datasets presented in this study can be found in online repositories. The names of the repository/repositories and accession number(s) can be found below: Research Data Center of the IQB, http://doi.org/10.5159/IQB_BiLieF_v1.

## Ethics Statement

Ethical review and approval was not required for the study on human participants in accordance with the local legislation and institutional requirements. The patients/participants provided their written informed consent to participate in this study.

## Author Contributions

JG: conceptualization, methodology, formal analysis, resources, data curation, writing—original draft, writing—review and editing, and visualization. PN: conceptualization, data curation, writing—review and editing, investigation, resources, and project administration. JS: writing—review and editing. All authors contributed to the article and approved the submitted version.

## Conflict of Interest

The authors declare that the research was conducted in the absence of any commercial or financial relationships that could be construed as a potential conflict of interest.

## Publisher’s Note

All claims expressed in this article are solely those of the authors and do not necessarily represent those of their affiliated organizations, or those of the publisher, the editors and the reviewers. Any product that may be evaluated in this article, or claim that may be made by its manufacturer, is not guaranteed or endorsed by the publisher.

## Footnotes

1
www.unipark.de
