# Supplementary material for: Teachers Between a Rock and a Hard Place: Goal Conflicts Affect Teaching Motivation Mediated by Basic Need Satisfaction
Source: Front Psychol. 2022 Jun 3;13:876521. doi: 10.3389/fpsyg.2022.876521 (PMC9204143; doi:10.3389/fpsyg.2022.876521)
Supplement: Supplementary file 1 [file Table_1.DOCX]

Supplementary Material

# Appendix

*Self-developed measure to assess the frequency of experiencing resource-based and inherent goal conflict*

| No. | Original instructions and items (German) | English translation by authors |
| --- | --- | --- |
|  | Lehrer/innen haben in ihrem Beruf vielfältige und höchst komplexe Aufgaben zu bewältigen. Wir möchten von Ihnen erfahren, wie Sie diese Anforderungen erleben und inwiefern Sie sich dadurch belastet fühlen.  Die Antwortskala ist zweigeteilt: Bitte geben Sie auf der linken Seite an, wie häufig Sie sich zwischen den beschriebenen Anforderungen hin und hergerissen fühlen. Auf der rechten Seite geben Sie bitte an, als wie stark Sie die Belastung dadurch empfinden. Bitte ersetzen Sie in Gedanken die "..." durch die jeweils angegebenen Anforderungen/Ziele.  Wie häufig kommt es vor, dass Sie sich dabei hin- und hergerissen fühlen zwischen… | Teachers have to cope with a wide range of highly complex tasks in their profession. We would like to hear from you how you experience these demands and to what extent you feel burdened by them.  The response scale is divided into two parts: On the left-hand side, please indicate how often you feel torn between the described requirements. On the right-hand side, please indicate how much of a burden this is for you. Please replace the “…” in your mind with the requirements/goals indicated in each case.  How often does it happen that you feel torn between… |
|  | Ressourcenorientierte Zielkonflikte | Resource-based Goal Conflicts |
| 1 | …der Unterrichtsvorbereitung und anderen Tätigkeiten? | …lesson preparation and other activities? |
| 2 | …der Teilnahme an Fort- und Weiterbildungsaktivitäten und anderen Tätigkeiten? | …participation in training and development activities and other occupations? |
| 3 | …der (Mit-) Gestaltung des Schullebens (z.B. Sommerfest, Weihnachtsfeier) und anderen Tätigkeiten? | …the (co-) organization of school life (e.g. summer festival, Christmas party) and other activities? |
| 4 | …der Pflege von Elternkontakten und anderen Tätigkeiten? | …maintaining parental contacts and other jobs? |
| 5 | …der (Vorbereitung von Aufgaben zur) individuellen Förderung von Schüler/innen außerhalb der Unterrichtszeit (z.B. im Förderunterricht) und anderen Tätigkeiten? | …the (preparation of tasks for) individual support of students outside of class time (e.g. in remedial classes) and other jobs? |
| 6 | …der Betreuung der Schüler/innen außerhalb der Unterrichtszeit (z.B. in den Pausen, in AGs) und anderen Tätigkeiten? | …the supervision of students outside of class time (e.g. during breaks, in school groups) and other tasks? |
|  | Inhärente Zielkonflite | Inherent Goal Conflicts |
| 1 | …dem Ziel, eine/n Schüler/in zu fördern und dem Bestreben, ihn/sie zu fordern? | …the goal of supporting a student and striving to further him/her? |
| 2 | … dem Ziel, zu einem/r Schüler/in Vertrauen aufzubauen und dem Bestreben, eine professionelle Distanz zu wahren? | …the goal of building trust with a student and the desire to maintain a professional distance? |
| 3 | …dem Ziel alle Schüler/innen gleich zu behandeln und dem Bestreben, einzelne Schüler/innen individuell zu fördern? | …the goal of treating all students equally and the effort to support individual students? |
| 4 | …dem Ziel, alle Schüler/innen zu guten Leistungen zu bringen und dem Bestreben, besonders gute Schüler/innen weiter zu fördern? | …the goal of getting all students to perform well and the effort to further support especially good students? |
| 5 | …dem Ziel, den Schüler/innen viel Freiraum zu lassen und dem Bestreben, Ordnung und Disziplin im Klassenzimmer zu haben? | …the goal of giving students a lot of freedom and the desire to have order and discipline in the classroom? |
| 6 | …dem Ziel, jede/n Schüler/in als Individuum anzusehen und dem Bestreben, in Schüler/innen bekannte Typen oder Muster (wieder) zu erkennen? | …the goal of viewing each student as an individual and the effort to (re)identify familiar types or patterns in students? |
| 7 | ...dem Ziel, durch Ihr Einwirken etwas Gutes zu bewirken und dem Bestreben, nichts falsch zu machen? | …the goal to bring about something good through your influence and the ambition to do nothing wrong? |
| 8 | … dem Ziel, sich mit den Schüler/innen auf Augenhöhe zu bewegen und dem Bestreben, für die Schüler/innen eine Autoritätsperson zu sein? | …the goal of being at eye level with the students and the aspiration to be an authority figure for the students? |
